# Screening of Leptin-LepR modulators using molecular docking and binding assay

**DOI:** 10.6026/973206300200404

**Published:** 2024-05-31

**Authors:** Albertana Jiménez-Pineda, José Luis Vique-Sánchez, Oscar Medina-Contreras, Claudia G Benitez-Cardoza

**Affiliations:** 1 Laboratorio de Investigación Bioquímica y Biofísica Computacional, ENMyH, Instituto Politécnico Nacional, Guillermo Massieu Helguera, No. 239, Fracc. "La Escalera", Ticomán, C.P. 07320, Ciudad de México, México; 2 Facultad de Medicina Mexicali, Universidad Autónoma de Baja California, CP 02100, BC, México; 3 Unidad de Investigación Epidemiológica en Endocrinología y Nutrición, Hospital Infantil de México Federico Gómez, CP 06720, Ciudad de México

**Keywords:** Leptin receptor, drug-like compounds, protein-compound docking, interactions, ADMET properties

## Abstract

Leptin is a pleiotropic hormone which, upon binding to its cognate leptin receptor (LepR), induces the activation of the JAK2/ERK, STAT3/STAT5 and IRS/PI3 kinase signaling cascades. Hence, we used molecular docking and a chemical library to identify 18 compounds with high probability of interacting with the leptin binding domain (LBD) of LepR. 6 out of 18 compounds were selected based on toxicological and physicochemical properties to evaluate their effect in the formation of Leptin-LepR complex using ELISA assays. The six compounds showed discreet but significant modulation on the complex formation. These results have important implications in proposing novel strategies for modulating the formation of the Leptin-LepR complex, as therapeutic alternatives for patho-physiologies where the formation of this complex is deregulated.

## Background:

The maintenance of homeostasis and the regulation of growth are essential physiological processes requiring precise control mechanisms. Among these mechanisms, the leptin-leptin receptor (LepR) axis plays a critical role in a wide array of physiological functions. Leptin, classified as a class I cytokine and its receptor, LepR, exert their influence on diverse processes. Leptin modulates food intake and energy expenditure by acting on anorexigenic pathways within the hypothalamus and regulating metabolism and energy homeostasis in peripheral tissues [1]. Additionally, the leptin-LepR axis participates in glucose homeostasis, reproductive functions, bone formation and wound healing processes. Leptin plays a significant role in both innate and adaptive immunity. Leptin controls the activation and function of macrophages, neutrophils, monocytes, dendritic cells and natural killer cells. Additionally, it promotes the production of pro-inflammatory cytokines. Also, leptin influences thymic and splenic homeostasis, promotes the proliferation of naive CD4+ T cells and skews T helper cell differentiation towards TH1 and TH17 responses while suppressing regulatory T cells [2]. Due to its involvement in immune system modulation, the leptin-LepR axis has been implicated in the development and progression of several autoimmune diseases, including multiple sclerosis, antigen-induced arthritis, hepatitis, colitis and glomerulonephritis. This link is further supported by evidence from animal models, where leptin-deficient rodents exhibit protection against these autoimmune conditions, while leptin administration restores disease susceptibility [3, 4]. Other evidence of the importance of the leptin/LepR axis in pathologies are the reports of higher expression levels of both leptin and its receptors in various types of cancer, such as prostate and breast cancer; specifically in breast cancer these increases have been significantly related to distant metastasis, other types of cancer where overexpression of leptin and leptin receptors has been observed is endometrial cancer and gastric cancer [5]. Six isoforms of LepR, generated by alternative splicing, have been described. These isoforms are classified into short (LepRa, LepRc, LepRd, LepRf) long (LepRb) and secreted (LepRe). All of them share the extracellular leptin-binding domain (LBD) of 816 amino acids, a transmembrane domain of 34 amino acids (except the soluble isoform). The cytoplasmic domains are different in each of the isoforms. The extracellular region is divided into six domains: an N-terminal domain (NTD), two CRH domains (CRH1 and CRH2), an immunoglobulin-like domain (IGD) and two additional fibronectin type III domains near the membrane (FN III) [6]. It has been described that LepR activation requires the CRH2, IGD and FN III domains. Only the CRH2 domain is strictly required for leptin binding to its receptor. This was demonstrated by measuring the KD of leptin binding to this domain, which was comparable to the KD of leptin binding to the entire extracellular domain of LepR. For this reason, this domain is commonly known as the leptin-binding domain. Through a detailed mutagenesis exploration of the CRH2 domain to determine the leptin/LepR interaction, the importance of five hydrophobic residues (503-IFLL-506, Y472) located in this domain has been evidenced; mutations of these residues lead to a decrease of leptin binding and decreased signaling [7]. Genetic evidence has indicated that the biological effects of leptin are primarily the result of binding to LepRb, which is the only splice variant of the leptin receptor that harbors all the residues necessary for signal transduction. LepRb is mainly expressed in the brain and in hematopoietic and immune cells. It has also been found highly expressed in the intestine, while the other splice variants show a wide distribution in tissues including kidney, ovary, uterus, testis, liver, lung, adrenal gland and spleen. The extensive expression of leptin receptor isoforms at the functional level denotes the pleiotropic effects of leptin [2]. Therefore, it is of interest to describe the screening of Leptin-LepR modulators using molecular docking and binding assay.

## Materials and Methods:

## Leptin receptor model:

The three-dimensional model of the leptin-binding domain (CRH2, residues 431 to 634) of the human leptin receptor (UniProt: P48357), was built using the I-TASSER server, as described by López-Hidalgo *et al.* [8]. Upon validation, the model was subjected to molecular dynamics simulations for 100 ns at 310 K using the high-performance program GROMACS [9]. The conformations were grouped in clusters according to their root-mean-square deviation of C-alpha-atomic positions values. The average conformation corresponding to the most populated cluster was used as the 3-D model in this work.

## Screening library:

For molecular coupling, the EXPRESS-pick collection stock of the Chembridge Corp. small molecule selection library was used [10]. The compounds of this library (Figure 1a, b, c - see PDF) fulfill the pharmacological properties of Lipinski's rules [11].

## Molecular docking:

Molecular Operating Environment (MOE) software was used for coupling, protonation and energy minimization of the PDB file with the default parameters (Placement: Triangle Matcher, rescoring 1: London ΔG, Refinement: Force field, rescoring 2: London ΔG, for all compounds were generated and stored 30 conformations) and the CHARMM27 force field [12]. During docking, the receptor remained rigid, while the ligand atoms were released to move to a maximum number of rotating bonds. All crystallographic water molecules were removed from the initial structure. The docking was site directed by selecting the region of interaction with leptin in LBD Y472, I503, F504, L505 y L506 [13].

## Free binding energy (ΔG_binding_):

The binding affinity of each complex (ligand-protein) was calculated, with the enthalpic contribution to the free energy of binding using a linear function using MOE [14]. The non-bonded interaction energies between the ligand and protein molecule involve Coulomb electrostatic interactions, Van der Waals and implied solvent interaction energies.

## Selection of compounds:

18 compounds were selected using the results of up to 30 conformers of each compound for selection. The value calculated for the free energy of binding (ΔG_binding_) of each complex (Ligand-Protein) was considered as a selection criterion, as reported [2, 5]. With these results, the best ΔG_binding_ averages were determined, as well as the standard deviation of each compound using the Excel software.

## Pharmacokinetic properties of compounds:

Description of chemical properties, hard-boiled egg and theoretical toxicity (carcinogenicity and mutagenicity) were calculated using SwissADME [15], admetSAR [16] and ADMETlab [17] and ProTox-II [18] web servers. For all calculations, the SMILES chain (Simplified Molecular-Input Line-Entry System) of each compound was inserted in every server.

## Bioactive Leptin ELISA assay:

Six compounds were tested to evaluate their intervention in the binding of leptin to the leptin receptor. Leptin W100E (obtained as described by Chimal-Vega *et al.* [19] and compounds A8, A9, A10, A11, A14 and A17 were used with the Bioactive Leptin ELISA kit from ALPCO, Catalog Number: 22-BLEPHU-E01, Version: 19.12.2022 Revision 003 - ALPCO 1.0, following the manufacturer's instructions. Before the assay, the corresponding wells of the plate were incubated for 2 hours with 1 mM of each compound, at room temperature and constant stirring at 350 rpm.

## Results:

## Selection of compounds using docking:

The binding site considered in this work, involves residues Y472, I503, F504, L505 and L506 of LBD-LepR, which have been reported as the most important site leptin. The 18 top poses, out of almost 500,000 compounds from Chembridge library, were selected, according to were the lowest ΔG_binding_ values (average of the ΔG_binding_ corresponding to 30 conformers per compound), as well as the standard deviation. The selected compounds were named, for convenience; with arbitrarily generated identification codes with letter A and a consecutive number (A1-A18). Table 1, shows the identification numbers of each compound, the calculated binding scores, the residues within LBD-LepR that interact with each molecule, as well as some physicochemical properties.

## Physicochemical properties of the compounds:

Table 1 presents the chemical names, smile code and some physicochemical properties of the selected compounds. Physicochemical properties, like lipophilia (WLogP), solubility (LogS) and polarity (tPSA; Å^2^), among others are determinant of the pharmacokinetic and pharmacodynamic processes, as well as the safety of the drugs [20].

## Pharmacokinetics and pharmacodynamics:

Further analyses of the 18 selected compounds were carried out to describe their pharmacokinetic and pharmacodynamic processes, using three website servers; SwissADME, admetSAR and ADMETlab, described in Table 2. Gastrointestinal absorption and access to the brain are two crucial behaviors for estimating at various stages of drug discovery processes. Considering the relationship between WLogP and tPSA values it is possible to accurately predict the passive human gastrointestinal absorption (HIA) and blood-brain barrier (BBB) permeability (Table 1, Figure 2a - see PDF). According to this model 3 out of the 18 molecules (A3, A13 and A16) might not be well absorbed by these pathways while four molecules are permeable through the blood-brain barrier (A8, A9, A10 and A11).

When evaluating the Distribution, two values were considered: the probability of each compound as substrate of permeability glycoprotein (P-gp) and the percentage of plasma protein binding (PPB). Five out of 18 compounds (A5, A8, A9, A10 and A11) might be P-pg substrate and four compounds showed PPB percentage equal to or greater than 90% (A6, A12, A16 and A17) (Table 2). P-gp belongs to the ABC superfamily of transporters and is also known as multidrug resistance protein (MDR). P-gp alters drug absorption (*e.g.*, by expelling drug molecules back into the gastrointestinal lumen in oral drug administration), distribution (*e.g.*, by preventing drug penetration into the brain), metabolism (*e.g.*, acting synergistically with cytochrome P450 3A) and excretion (*e.g.*, by affecting the functions of the biliary and renal tubules) [21]. This suggests that compounds A5, A8, A9, A10 and A11 that are predicted as substrates of P-gp may exhibit poor bioavailability by impeding their permeability through physiological barriers.

Another factor which significantly influences the distribution and elimination of drugs is plasma protein binding. A percentage of the circulating drug can bind with various affinities to blood proteins and according to the hypothesis of the free drug, only the free drug is available to act at physiological sites of action; serum albumin, lipoproteins and acid glycoprotein alpha-1 (AAG) are the main plasma proteins involved in sequestration [22]. The 18 compounds evaluated are predicted with more than 50% plasma protein binding (Table 2) and particularly, compounds A6, A12, A16 and A17 are predicted with less than 10% as a free fraction. However, 45% of FDA-approved drugs are classified as highly bound (>95% PPB) [23] so it is likely that our compounds under physiological conditions may have a biological effect.

The possible interaction of drugs with isoenzymes of the cytochrome superfamily P450 (CYP) is fundamental to determine their metabolism. Therefore, the possible inhibition of the five main isoforms (CYP1A2, CYP2C19, CYP2C9, CYP2D6, CYP3A4) was evaluated, in none of the cases is a single molecule expected to be an inhibitor of these five isoforms (Table 2). The excretion of the compounds was addressed with the prediction of the half-life (T1/2) and the renal clearance (CL). All the poses showed low range values for both T1/2 and CL (Table 2).

## Toxicological properties:

The toxicological safety of the compounds, analyzed by the PROTOX II server is shown in Figure 2b. The heat map shows prediction of hepatotoxicity, where 3 of the compounds are predicted with moderate risk (A2, A3 and A16), while in carcinogenicity (Figure 2b - see PDF) the compounds A1, A2, A16 and A17 are likely at risk. Another important aspect that we considered was immunogenicity where for 6 of the 18 cases we found irrigation (Figure 2b - see PDF); as for the mutagenicity parameter, the server found risk in 5 molecules (Figure 2b - see PDF). It is interesting to note that for none of the structures evaluated is predicted risk of cytotoxicity (Figure 2b - see PDF). In addition, the mean lethal dose (LD50) and the classification of the compounds into toxicity classes, defined according to the globally harmonized system of chemical labeling classification (GHS) [24] are shown in Table 2. Three compounds lie in category 3 (A1, A13 and A17) with LD50 >50 mg/kg ≤300, only the A14 compound is classified in category 5 with an LD50 >5000 mg/kg and the rest of the compounds were classified in category 4 (LD50 >300 mg/kg ≤2000) (Table 2).

## Bioactive Leptin binding affinity:

From the results of the sections above, we selected six compounds (A8, A9, A10, A11, A14 and A17), which showed the best ADMET properties and low toxicity to confirm their potential interaction to LepR. A competitive binding assay was conducted. The Bioactive Leptin ELISA kit (ALPCO) was utilized, employing W100E leptin [19]. The assay measured the amount of W100E leptin bound to the immobilized leptin receptor on the kit plate after pre-incubation with each compound. The results demonstrate that compound A8 discreetly but significantly inhibit W100E leptin binding about 7.89%, while the rest of compounds exhibited varying degrees of enhanced binding. Notably, compound A9 significantly increased bound leptin by 12.93% (Figure 2c - see PDF). These findings suggest that the tested compounds possess affinity for the leptin receptor and can modulate the amount of leptin binding to the receptor.

## Discussion:

In the current study, we searched for compounds with binding affinity to Y472, I503, F504, L505 and L506 residues already reported as the amino acids that make up the LBD in the leptin receptor. In modern drug discovery, protein-ligand docking plays an important role in predicting the orientation of the ligand when it is bound to a protein receptor or enzyme using shape and electrostatic interactions to quantify it. The van der Waals interactions also play an important role, in addition to electrostatic interactions and the formation of hydrogen bonds. The sum of all these interactions is approximated by a docking score, which represents potentiality of binding [23]. The docking was site directed and the amino acids selected as the binding site of the compounds formed the LBD. However, due to protein folding, several other amino acids can interact with the compounds found and tested, as shown in Table 1, the amino acids that according to docking interact with all compounds are R615 and L505, with only L505 being part of the LBD, for Y472 an interaction is predicted with 5 of the 18 compounds (A6, A8, A9, A10 and A11), for I503 it is predicted to be an affinity site for 12 of the compounds (A1, A3-A6, A12-18) , with possible less participation, F504 is predicted to be related to A13 and A16 and for L506 no interactions with the selected compounds are predicted. Natural leptin has two tryptophan residues (Trp 100 in loop III and Trp 138 at the C terminus of αD). The W100E variant of leptin (pseudo-WT) was made for crystallization purposes and has since been shown to have similar biological activity to the WT [24]. In our bioactive leptin assay results, the binding affinity of leptin W100E to its receptor was modified with the 6 compounds tested, interestingly only compound A8 decreased the amount of leptin detected bound to the receptor suggesting that this compound could be an antagonist of leptin signaling, while compounds A9, A10, A11, A14 and A17 could act as agonists.

## Conclusions:

Potential modulators of the leptin-LepR interaction were identified using molecular docking. Eighteen compounds exhibiting the lowest binding free energy (ΔG_binding_) values were selected for further investigation, suggesting a high probability of interaction with the LepR-LBD. The six compounds demonstrating the most favorable binding poses (A8, A9, A10, A11, A14 and A17) were evaluated in vitro using ELISA assays to quantify their impact on Leptin-LepR complex formation. Compound A8 demonstrated a notable reduction in Leptin-LepR interaction by over 7%. Conversely, the remaining five compounds (A9, A10, A11, A14 and A17) enhanced complex formation, with increases ranging from 2% (A14) to 13% (A9). These findings provide valuable insights into the molecular mechanisms governing Leptin-LepR interactions and offer potential avenues for the development of novel therapeutic strategies to address leptin resistance.

## Figures and Tables

**Table 1:** Chemical details, docking scores and physicochemical properties of 18 selected compounds

**Docking**						**Physicochemical properties**			
**Compound ID Chembridge Corporation**	**Chemical name**	**Smile code**	**Interacting amino acid residues**	**Least ΔG_binding_ (kcal mol-1)**	**Average ΔG_binding_ ± SD (kcal mol-1)**	**Molecular weight (g mol-1)**	**WLogP**	**LogS**	**tPSA (Å^2^)**
A1. 7788812	Ethyl 2-{[(3-nitrophenyl) amino] carbonyl} hydrazinecarboxylate	[N+](=O)([O])C1=CC=CC(=C1)NC(=O)NNC(OCC)=O	R615, L442, I503, L505, D532, N566	-12	-11.02 ± 0.40	268	1.19	-2.12	125.28
A2. 7790013	Tert-butyl 2-{[(4-chlorobenzyl) amino] carbonothioyl} hydrazinecarboxylate	C(NCC1=CC=C(C=C1)Cl)(=S)NNC(=O)OC(C)(C)C	R615, Y441, L442, L505, D532, F563, P564, N566	-13.14	-11.13 ± 0.65	316	2.59	-3.38	94.48
A3. 9037526	7-(3,4-dimethoxyphenyl)-2-hydroxy-9 (trifluoromethyl) pyrido [2',3':4,5] thieno[2,3-b] pyridin-4(1H)-one	C12NC(O)=CC(=O)C=1SC1N=C(C3=CC=C(C(OC)=C3)OC)C=C(C(F)(F)F)C2=1	T438, L442, T443, Q501, I503, L505, P531, D532, V535, N566, R615	-14.59	-11.08 ± 1.14	422	5.7	-5.53	112.68
A4. 9038033	9-ethyl-4-hydroxy-7,8,9,10-tetrahydropyrido [2',3':4,5] thieno [2,3-b] quinolin-2(1H)-one	C12NC(=O)C=C(O)C=1SC1=NC3CCC(CC)CC=3C=C21	L442, I503, L505, V535, N566, R615	-14.71	-11.25 ± 1.22	300	3.36	-4.01	94.22
A5. 5254870	4-[4-(benzyloxy) phenyl]-5-[2-(4-methyl-1-piperazinyl) ethyl]-2,4-dihydro-3H-1,2,4-triazole-3-thione	N(C1=CC=C(C=C1)OCC1C=CC=CC=1)1C(CCN2CCN(CC2)C)=NNC1=S	D532, L442, T443, Q501, I503, L505, P564, N566, R615	-13.15	-11.14 ± 0.82	410	2.39	-4.1	81.41
A6. 7807483	N-(2,4-difluorophenyl)-N'-1-naphthyl-1,2-hydrazinedicarbothioamide	C(=S)(NNC(=S)NC1C=CC(=CC=1F)F)NC1=CC=CC2C=CC=CC1=2	R615, T438, L442, Y472, I503, L505, P531, D532, G618, L619	-12.5	-11.10 ± 0.75	388	4.76	-5.06	112.3
A7. 5697901	N-cyclohexyl-2-(3,4,5-trihydroxybenzoyl) hydrazinecarboxamide	C(O)1=C(O)C=C(C=C1O)C(=O)NNC(=O)NC1CCCCC1	L442, D532, R615, T438, L505, P531	-14.95	-11.41 ± 1.03	309	1.08	-2.37	130.92
A8. 5803718	N-{2-[2-(5-isopropyl-2-methylphenoxy) ethoxy]ethyl}-1,2-ethanediamine oxalate	C1=C(OCCOCCNCCN)C(C)=CC=C1C(C)C	D532, R615, L442, Y472, L505, S433, P564, N566, G618, L619	-15.18	-11.45 ± 1.17	280	2.06	-2.32	56.51
A9. 5803816	N-{2-[2-(2-chloro-6-methylphenoxy) ethoxy]ethyl}-1,2-ethanediamine oxalate	C(OCCOCCNCCN)1=C(C)C=CC=C1Cl	D532, R615,	-14.85	-11.15 ± 1.12	273	1.59	-2.05	56.51
A10. 5803882	N-{2-[2-(3-isopropylphenoxy) ethoxy] ethyl}-1,2-ethanediamine oxalate	C1(=CC=CC(OCCOCCNCCN)=C1)C(C)C	D532, R615, L442, Y472, L505, P564, N566, R615, G618, L619	-15.83	-11.18 ± 1.1	266	1.75	-2.01	56.51
A11. 5805016	N-{2-[2-(mesityloxy) ethoxy] ethyl}-1,2-ethanediamine oxalate	C(OCCOCCNCCN)1C(C)=CC(=CC=1C)C	D532, R615, Y472, L505, E565, G618, L619	-14.35	-11.09 ± 1.21	266	1.56	-2.06	56.51
A12. 5151565	3,3'-(1,1-ethanediyl) bis (4-hydroxy-2H-chromen-2-one)	C(C(C)C1C(=O)OC2=C(C=CC=C2)C=1O)1C(=O)OC2=C(C=CC=C2)C=1O	R615, T438, L442, I503, L505, P531, D532, V535, N566	-13.12	-11.25 ± 0.93	350	3.46	-3.75	100.88
A13. 5152582	4,4'-sulfonyldiphthalic acid	S(=O)(=O)(C1C=CC(=C(C(O)=O)C=1)C(O)=O)C1C=CC(=C(C(O)=O)C=1)C(O)=O	T438, R615, Y441, L442, I503, F504, L505, P531, F563, P564, N566	-13.54	-11.054 ± 0.79	394	2.39	-2.73	191.72
A14. 9230886	4-hydroxy-9-methyl-11-phenyl-7,8,9,10-tetrahydropyrido [2',3':4,5] thieno [2,3-b]-1,6-naphthyridin-2(1H)-one	C12C(C3C=CC=CC=3)=C3CN(C)CCC3=NC=1SC1C(=CC(=O)NC2=1)O	L442, T438, T443, I503, L505, P531, V535, N566, R615	-15.63	-11.14 ± 1.05	363	2.97	-4.17	97.46
A15. 9237042	9-ethyl-4-hydroxy-7,8,9,10- tetrahydropyrimido [4',5':4,5] thieno [2,3-b] quinolin-2(1H)-one	C12C(O)=NC(NC=1C1=C(N=C3C(CC(CC3)CC)=C1)S2)=O	N566, L442, T443, I503, L505, E565, R615	-14.18	-11.41 ± 0.97	301	2.75	-4.25	107.11
A16. 5301496	2,2'-[1,2,5-oxadiazole-3,4-diylbis(iminocarbonyl)] dibenzoic acid	C1(=NON=C1NC(=O)C1=CC=CC=C1C(O)=O)NC(=O)C1=CC=CC=C1C(O)=O	R615, T438, L442, I503, F504, L505, P531, D532, V535, P564, N566	-12.56	-11.44 ± 0.61	396	1.59	-2.92	171.72
A17. 5190805	bis(4-hydroxy-2-oxo-2H-chromen-3-yl) acetic acid	C(C(C(O)=O)C1C(=O)OC2=C(C=CC=C2)C=1O)1C(=O)OC2=C(C=CC=C2)C=1O	N566, R615, T438, L442, I503, L505, P531, D532, V535, F563, P564	-15.49	-12.54 ± 0.9	380	2.53	-3.35	138.18
A18. 9256439	9-ethyl-4-hydroxy-11-(trifluoromethyl)-7,8,9,10- tetrahydropyrimido [4',5':4,5] thieno [2,3-b] quinolin-2(1H)-one	C12C(O)=NC(NC=1C1=C(N=C3C(CC(CC3)CC)=C1C(F)(F)F)S2)=O	T438, L442, T443, I503, L505, P531, D532, V535, N566, R615	-14.45	-12.06 ± 0.98	369	4.92	-5.09	107.11

**Table 2:** ADMET profile of 18 selected compounds.

**Pharmacokinetic properties**							
**ID compound Chembridge Corp.**	**D (Distribution)**		**M (Metabolism)**		**E (Excretion)**		**Toxicity**
	**P-gp substrate**	**Plasma protein binding (PPB) (%)**	**Substrate**	**Inhibitor**	**Half time (T1/2) (h)**	**Renal clearance (CL) (ml/min/kg)**	**LD50 (toxicity class)**
A1. 7788812	No	54.776	CYP3A4		0.416	1.525	11000 mg/kg ^-4^
A2. 7790013	No	72.658	CYP3A4	CYP2C19	0.836	1.457	630 mg/kg ^-4^
A3. 9037526	No	88.8	CYP3A4	CYP1A2, CYP2C19, CYP2C9, CYP3A4	1.852	1.225	1000 mg/kg ^-4^
A4. 9038033	No	84.161	CYP3A4, CYP2C9	CYP1A2, CYP2C9	1.7	1.338	1000 mg/kg ^-4^
A5. 5254870	Yes	88.922	CYP3A4	CYP1A2, CYP2C19, CYP3A4	1.758	1.741	1000 mg/kg ^-4^
A6. 7807483	No	91.41	---	CYP1A2, CYP2C19, CYP2C9	2.066	1.011	2000 mg/kg ^-4^
A7. 5697901	No	57.84	---	---	0.624	1.458	2000 mg/kg ^-4^
A8. 5803718	Yes	66.536	CYPD6	---	1.442	2.201	350 mg/kg ^-4^
A9. 5803816	Yes	60.315	CYP3A4, CYPD6	CYP1A2, CYP2C19, CYP2D6	1.157	1.86	1750 mg/kg (^4^)
A10. 5803882	Yes	63.236	CYP3A4	---	1.41	2.255	610 mg/kg ^-4^
A11. 5805016	Yes	59.496	CYPD6	---	1.51	2.283	350 mg/kg ^-4^
A12. 5151565	No	93.146	CYP2C9	CYP2C9	2.022	1.864	233 mg/kg ^-3^
A13. 5152582	No	79.104	---	---	1.446	0.791	83 mg/kg ^-3^
A14. 9230886	No	88.631	CYP3A4	---	1.951	1.731	5000 mg/kg ^-5^
A15. 9237042	No	80.207	CYP3A4	CYP1A2 CYP2C9	1.417	1.347	1000 mg/kg ^-4^
A16. 5301496	No	90.513	---	CYP1A2	1.158	0.692	1450 mg/kg ^-4^
A17. 5190805	No	90.997	CYP2C9	CYP2C9	1.788	1.277	233 mg/kg ^-3^
A18. 9256439	No	85.334	CYP3A4	CYP2C9	1.763	1.247	1000 mg/kg ^-4^
Median lethal dose (LD50) and toxicity class.
Class 1 is fatal if swallowed (LD50 ≤ 5 mg/kg),
class 2 fatal if swallowed (LD50 > 5 mg/kg ≤ 50),
class 3 toxic if swallowed (LD50 > 50 mg/kg ≤ 300),
class 4 can be harmful if swallowed (LD50 >300 mg/kg ≤ 2000),
class 5 can be harmful if swallowed (LD50 > 2000 mg/kg ≤ 5000)
and class 6 non-toxic (LD50 > 5000 mg /kg).
